# Cleidocranial dysplasia with preserved function under conservative management: a case report

**DOI:** 10.3389/fped.2026.1844281

**Published:** 2026-08-11

**Authors:** Huijiao Xu, Junmei Ma, Xiaosong Huang, Siyu Pu, Fang Hou, Wenying Liu, Jiajun Chen

**Affiliations:** 1 Department of Pediatric Surgery, West China Second University Hospital, Sichuan University, Chengdu, China; 2 Key Laboratory of Birth Defects and Related Diseases of Women and Children (Sichuan University), Ministry of Education, Chengdu, China; 3 Department of Pediatric Surgery, School of Medicine, Sichuan Provincial People’s Hospital, University of Electronic Science and Technology of China, Chengdu, China; 4 Sichuan Provincial People’s Hospital East Sichuan Hospital & Dazhou First People’s Hospital, Dazhou, China

**Keywords:** cleidocranial dysplasia, conservative management, preserved function, recombinant human growth hormone, RUNX2

## Abstract

**Introduction:**

Cleidocranial dysplasia (CCD) is a rare autosomal dominant skeletal disorder caused by pathogenic variants in the RUNX2 gene and characterized by delayed closure of cranial sutures, clavicular hypoplasia, and dental abnormalities. The clinical phenotype is highly heterogeneous, and data on the long-term natural history of CCD with preserved function remain limited.

**Methods:**

We reviewed the longitudinal clinical data of a male child with genetically confirmed CCD carrying a RUNX2 c.631C > T (p.R211W) variant. CCD-specific evaluation was initiated at approximately 4 years of age because of persistent delayed anterior fontanelle closure and clavicular abnormalities. Assessments included physical examination, cranial CT/MRI, chest imaging, panoramic dental radiography, whole-exome sequencing with parental validation, and endocrine follow-up.

**Conclusion:**

This case supports individualized conservative management for selected patients with CCD who have preserved function despite structural abnormalities. Longitudinal multidisciplinary follow-up is essential for guiding dental, endocrine, orthopedic, and functional management.

**Discussion:**

Despite typical skeletal and dental manifestations, the patient maintained preserved shoulder and upper limb function through 12 years of age, without recurrent fractures, persistent pain, functional limitation, or need for orthopedic or thoracic surgical intervention. A function-oriented conservative management strategy remained appropriate. Height SDS improved during rhGH-related follow-up; however, this observation should be interpreted cautiously because growth may also have been influenced by hypothyroidism, thyroid hormone replacement, normal development, and pubertal maturation.

## Introduction

Cleidocranial dysplasia (CCD) is a rare autosomal dominant skeletal disorder with an estimated prevalence of approximately 1 per 1,000,000 individuals (1). The most commonly implicated causative gene is RUNX2 (2, 3). Patients with CCD exhibit highly heterogeneous clinical manifestations, including delayed closure of cranial sutures, clavicular hypoplasia, and abnormalities in tooth eruption, reflecting varying degrees of skeletal involvement (4, 5). The phenotypic spectrum of CCD is broad, ranging from severe cases involving multiple systems that may require repeated surgical interventions to mild presentations with relatively preserved function (6–8). However, longitudinal data regarding the natural history, functional outcomes, and growth trajectories of patients with genetically confirmed CCD, particularly those with preserved functional status despite typical skeletal manifestations, remain limited.

Here, we report a male child with genetically confirmed CCD caused by a RUNX2 c.631C > T (p.R211W) variant. The diagnosis was established at approximately 4 years of age based on characteristic clinical, radiological, and genetic findings. We describe his subsequent longitudinal clinical course, including imaging findings, functional outcomes, endocrine management, and growth trajectory through 12 years of age, to provide insights into the long-term multidisciplinary management of CCD.

## Case presentation

The patient was a male child born at term via spontaneous vaginal delivery, with no abnormalities in birth weight, length, or Apgar scores. There was no relevant family history, and both parents had no history of skeletal abnormalities, dental anomalies, or short stature.

The patient was initially followed because of hypothyroidism diagnosed at approximately 7 months of age and received regular levothyroxine replacement therapy. At approximately 4 years of age, persistent delayed anterior fontanelle closure, short stature, delayed bone age, and clavicular abnormalities prompted further radiological evaluation and genetic investigation for suspected cleidocranial dysplasia. After the diagnosis of CCD was confirmed, the patient underwent CCD-specific multidisciplinary follow-up through 12 years of age. The chronological clinical course is summarized in Table 1.

**Table 1:** Chronological timeline of the patient’s clinical course.

Age/period	Clinical event
7 months	Hypothyroidism was diagnosed, and levothyroxine replacement therapy was initiated.
7 months −4 years	During routine pediatric/endocrine follow-up, delayed closure of the anterior fontanelle was noted.
4 years	Radiological assessment and whole-exome sequencing confirmed CCD with a heterozygous RUNX2 c.631C > T (p.R211W) variant.
8 years	rhGH therapy was initiated because of persistent short stature and delayed bone age despite adequate thyroid hormone replacement. A left inguinal hernia was also treated by laparoscopic repair.
10 years	The anterior fontanelle was found to have clinically closed on physical examination during outpatient follow-up.
12 years	At the latest follow-up, the patient maintained preserved shoulder and upper limb function, with no recurrent fractures, persistent pain, functional limitation, or need for orthopedic, thoracic, orthodontic, or oral surgical intervention.

CCD, cleidocranial dysplasia; rhGH, recombinant human growth hormone.

Delayed closure of the anterior fontanelle was noted during early childhood based on clinical history. CCD-specific evaluation was initiated at approximately 4 years of age, when persistent delayed anterior fontanelle closure and clavicular abnormalities raised suspicion for CCD. Craniofacial examination showed persistent delayed fontanelle closure and mild frontal bossing. Mild pectus excavatum was also observed. Palpation of both clavicles revealed firm cartilage-like prominences at the midshaft regions without abnormal mobility, and the proximal and distal clavicular segments appeared shortened, consistent with bilateral clavicular pseudarthrosis. Shoulder range of motion was preserved, with no evident pain or muscle weakness. Neurological examination was unremarkable.

**Figure 1:**
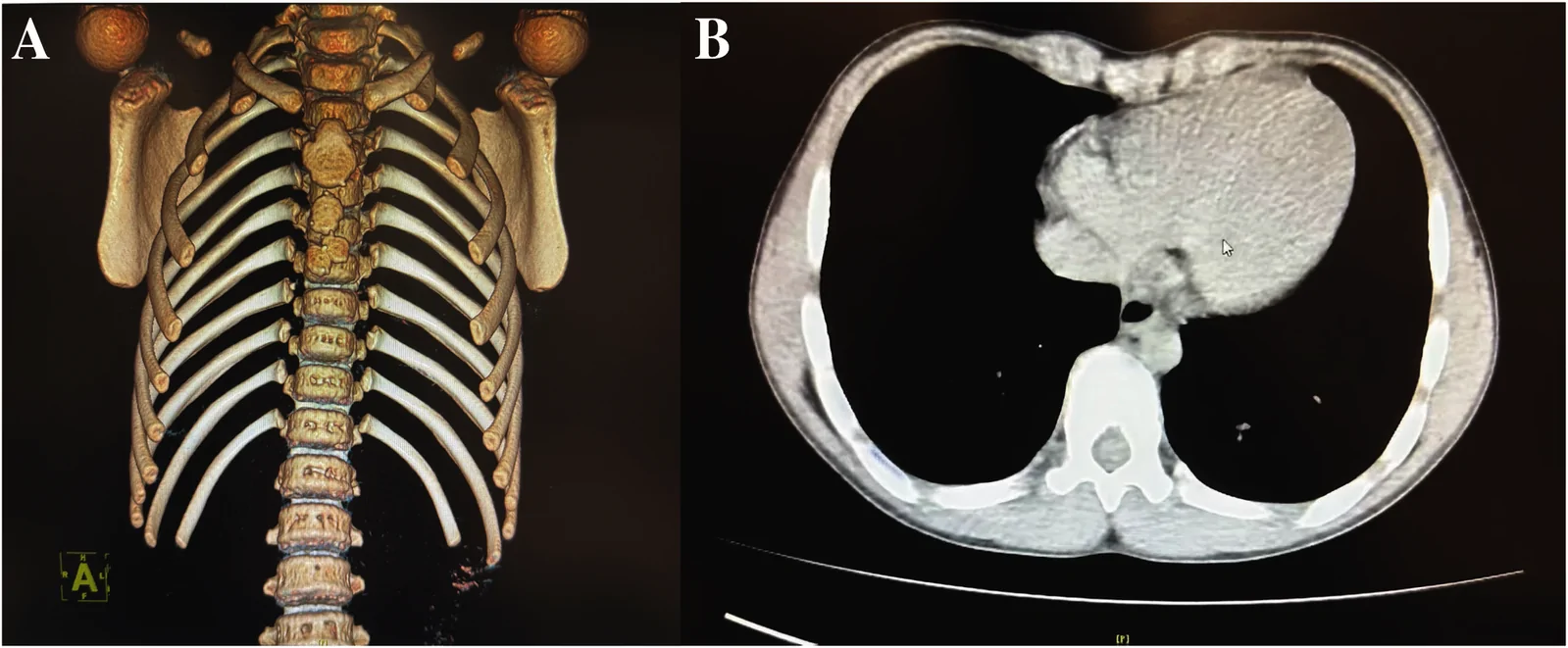
Radiological features of the chest of CCD. **(A)** Three-dimensional CT reconstruction demonstrates bilateral clavicular hypoplasia with segmental discontinuity, consistent with pseudoarthrosis. The clavicles appear shortened with dysplastic morphology, while the shoulder girdle remains relatively preserved. **(B)** Axial chest CT shows mild sternal depression without evidence of cardiopulmonary compression.

Serial cranial CT and MRI performed during early CCD-specific follow-up demonstrated delayed ossification of the skull and incomplete closure of cranial sutures, while brain parenchyma and pituitary morphology were normal. During subsequent clinical follow-up, the anterior fontanelle was found to have closed by approximately 10 years of age on physical examination. No neurological symptoms or functional impairment related to cranial abnormalities were observed thereafter. Chest radiographs and computed tomography during follow-up revealed bilateral clavicular hypoplasia with segmental discontinuity consistent with pseudarthrosis (Figure 1A), as well as mild pectus excavatum (Figure 1B), without abnormalities in the lungs or mediastinum. Lumbosacral spine radiographs showed no structural deformities.

**Figure 2:**
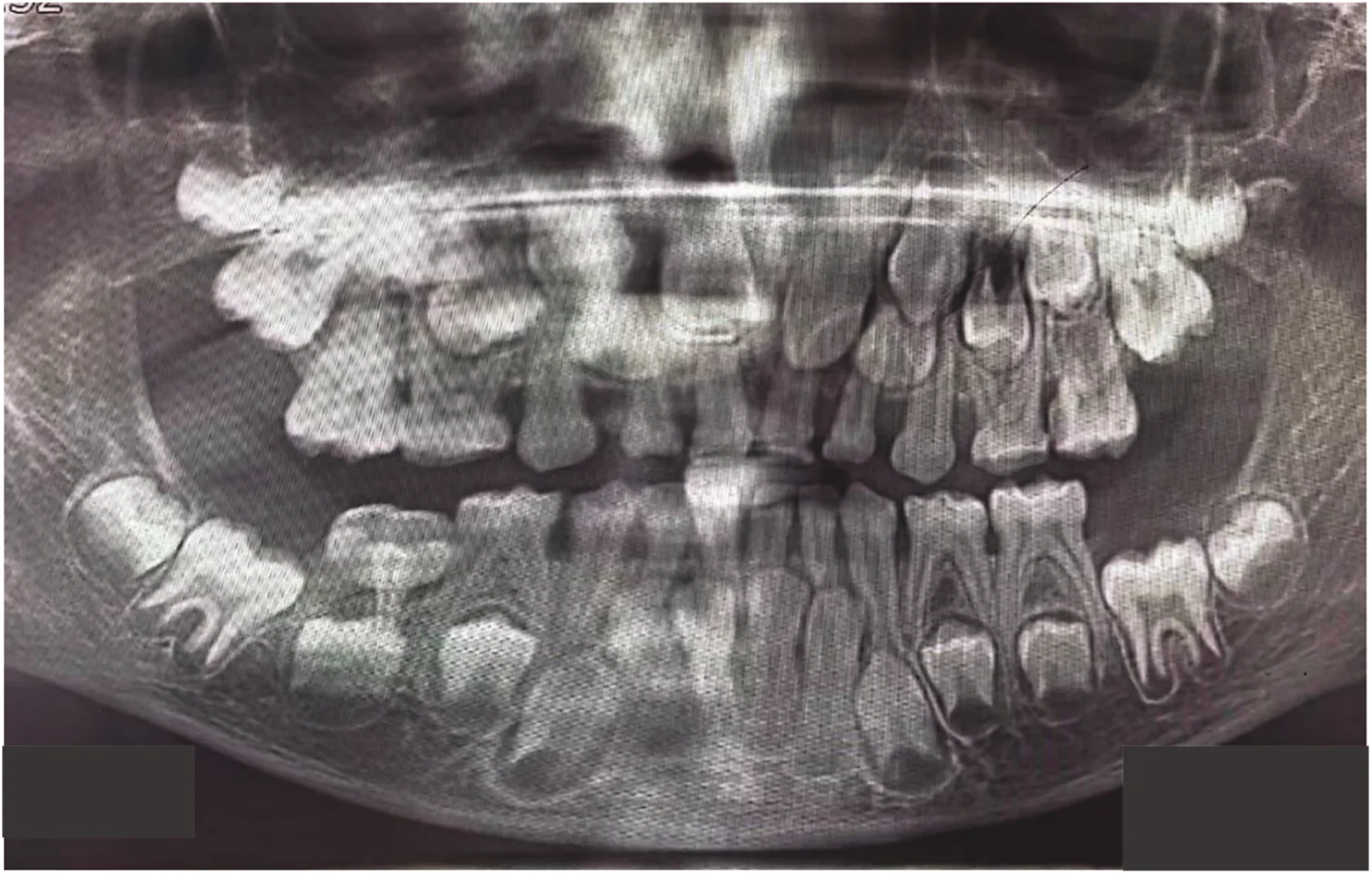
Panoramic radiograph of the dentition. The radiograph shows delayed exfoliation of primary teeth, delayed eruption of permanent teeth, and delayed dental development. Uneven development and abnormal positioning of several maxillary and mandibular teeth are observed. No obvious supernumerary teeth are identified on this radiograph.

A panoramic dental radiograph showed delayed exfoliation of primary teeth, delayed eruption of permanent teeth, and delayed dental development, consistent with characteristic dental manifestations of CCD (Figure 2). No obvious supernumerary teeth, impacted teeth requiring intervention, or other dental abnormalities requiring immediate treatment were identified on radiographic examination.

**Figure 3:**
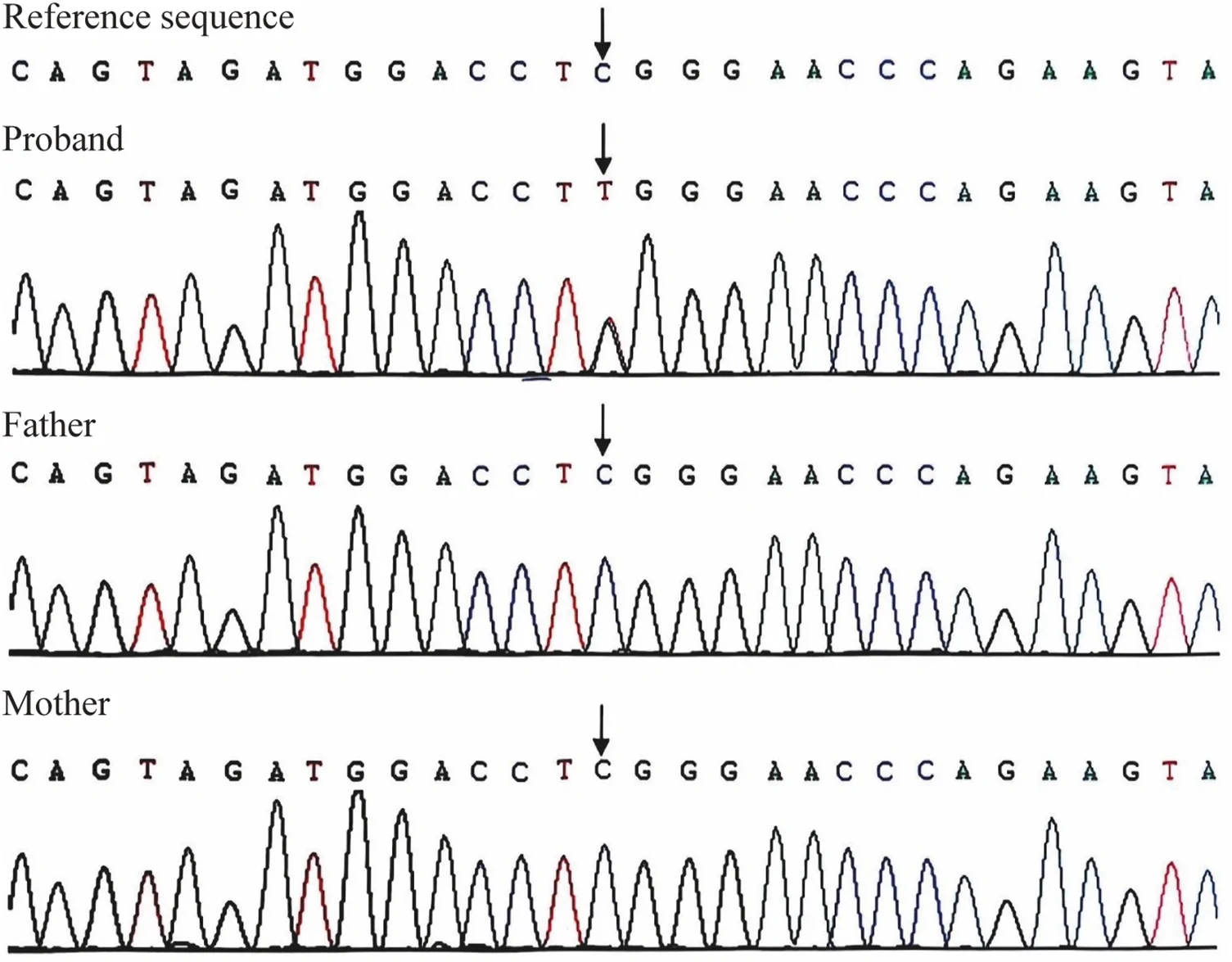
Sanger sequencing of the RUNX2 variant. Sanger sequencing identified a heterozygous c.631C>T (p.R211W) variant in the proband. The affected nucleotide is indicated by an arrow. The reference sequence shows the wild-type nucleotide (C), whereas the proband demonstrates overlapping peaks consistent with a heterozygous substitution (C>T). Both parents show the wild-type sequence, confirming that the variant occurred de novo.

Whole-exome sequencing performed at approximately 4 years of age identified a heterozygous RUNX2 c.631C > T (p.R211W) variant located within the Runt DNA-binding domain. Parental testing confirmed that neither parent carried this variant, suggesting a *de novo* occurrence (Figure 3). According to ACMG/AMP criteria, this variant was classified as pathogenic.

The patient was diagnosed with hypothyroidism at approximately 7 months of age and received regular levothyroxine replacement therapy. Thyroid function was monitored during follow-up and remained adequately controlled under replacement therapy.

Before recombinant human growth hormone (rhGH) therapy, pediatric/endocrine follow-up showed a persistently low height percentile and delayed bone age. At 6 years and 6 months of age, his height was 113.9 cm (height SDS, −1.44; 7.4th percentile), and his weight was 18.0 kg (weight SDS, −1.35; 8.8th percentile). At 7 years and 1 month of age, his height remained at a low percentile (116.1 cm; height SDS, −1.56; 5.9th percentile), with a documented height gain of 2.2 cm over the preceding approximately 6 months.

Because of persistent concern regarding growth trajectory and delayed bone age despite adequate thyroid hormone replacement, rhGH therapy was initiated at approximately 8 years of age after detailed counseling regarding potential risks and benefits. The treatment dose was 0.05 mg/kg per day, with regular monitoring of height, weight, growth velocity, bone age, thyroid function and other endocrine parameters. During rhGH-related follow-up, height SDS improved from −1.56 at 7 years and 1 month to −0.31 at 8 years and 7 months, −0.23 at 9 years and 2 months, and +0.09 at 12 years and 1 month. No significant adverse effects related to rhGH therapy were documented.Representative growth parameters before and during rhGH-related follow-up are summarized in Table 2. At approximately 8 years of age, a left indirect inguinal hernia was identified and treated by laparoscopic repair, with good postoperative recovery.

**Table 2:** Representative growth parameters before and during rhGH-related follow-up.

Age	Height	Height SDS	Height percentile	Weight	Weight SDS	Treatment status
6 years 6 months	113.9 cm	−1.44	P7.4	18.0 kg	−1.35	Before rhGH; receiving levothyroxine replacement therapy
7 years 1 month	116.1 cm	−1.56	P5.9	19.5 kg	−1.16	Before rhGH; receiving levothyroxine replacement therapy
8 years 7 months	129.7 cm	−0.31	P37.8	24.35 kg	−0.69	During rhGH-related follow-up
9 years 2 months	132.8 cm	−0.23	P40.9	27.4 kg	−0.38	During rhGH-related follow-up
12 years 1 month	150.5 cm	0.09	P53.5	37.6 kg	−0.4	Latest follow-up

rhGH, recombinant human growth hormone; SDS, standard deviation score.

**Figure 4:**
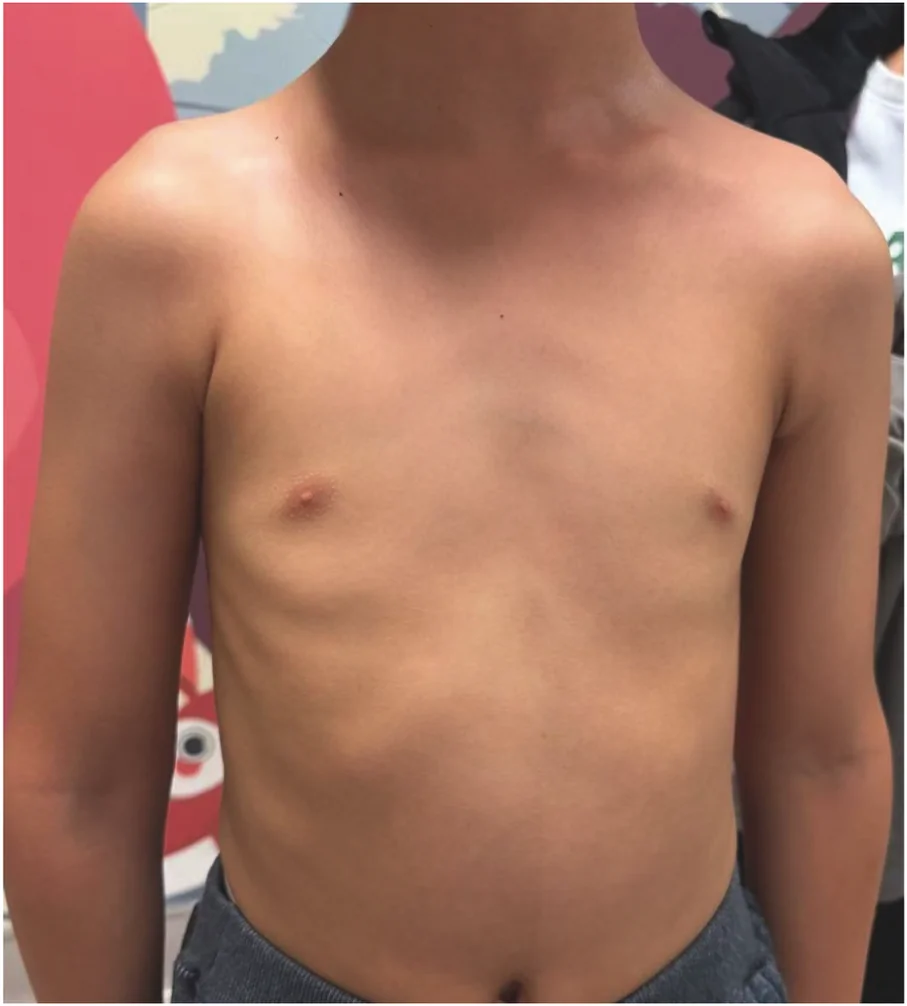
Clinical appearance of the chest wall. Anterior chest photograph demonstrating mild pectus excavatum and bilateral clavicular contour abnormalities. We believe these captions accurately describe the content of each figure. Please let us know if any further adjustments are needed.

At the latest follow-up at 12 years of age, the patient remained under multidisciplinary follow-up (Figures 4). Dental surveillance was continued for delayed exfoliation of primary teeth, delayed eruption of permanent teeth, and delayed dental development, without the need for orthodontic or oral surgical intervention to date. Shoulder and upper limb function remained well preserved, and the patient was able to participate in daily and sports activities, including basketball and swimming. No recurrent fractures, persistent pain, functional limitation, neurological symptoms, or impairment in activities of daily living were observed. Cognitive and academic development were normal. Given the preserved functional status and absence of progressive symptoms, no clavicular reconstruction or pectus excavatum correction was performed, and a function-oriented conservative management strategy was continued.

## Discussion

CCD is a rare autosomal dominant skeletal disorder primarily caused by pathogenic variants in RUNX2 located on chromosome 6p21 (2). RUNX2 encodes a key transcription factor of the RUNX family and serves as a master regulator of both intramembranous and endochondral ossification by controlling osteoblast differentiation and bone matrix formation (9). Reduced functional activity of RUNX2 leads to impaired ossification of multiple skeletal sites, including the skull, clavicles, thorax, and dentition, which constitutes the principal pathogenic mechanism of CCD (10). Previous studies have proposed a “RUNX2 dosage/threshold effect”,demonstrating that a minimum level of RUNX2 activity is required for normal skeletal development. In hypomorphic mouse models, RUNX2 expression needs to be maintained at approximately 70% of wild-type levels to ensure normal ossification, whereas further reduction results in classical CCD features such as delayed cranial suture closure, clavicular hypoplasia, and supernumerary teeth. In contrast, relatively preserved RUNX2 function may be associated with milder skeletal abnormalities (10).

In the present case, the RUNX2 c.631C > T (p.R211W) variant is a missense mutation located within the Runt homology domain (RHD), which is responsible for DNA binding and interaction with CBF-*β*. Functional studies of CCD-associated variants have shown that missense mutations within the RHD often impair DNA binding and transcriptional activity, including variants at or near codon 211, supporting the pathogenic relevance of this mutation (11, 12). However, this case further supports the notion that even well-established pathogenic RUNX2 variants may exhibit considerable phenotypic heterogeneity (10). Although the patient presented with several characteristic skeletal manifestations of CCD, he maintained preserved functional status throughout long-term follow-up without requiring orthopedic intervention. This observation suggests that factors beyond the underlying genetic variant, including endocrine status and other biological or environmental influences, may contribute to the variability of clinical expression.

Clavicular hypoplasia or aplasia is a hallmark feature of CCD, and some patients may develop unilateral or bilateral clavicular pseudarthrosis (4). Previous reports have largely focused on severe cases with prominent deformities, functional impairment, pain, or recurrent fractures, in which surgical interventions such as clavicular reconstruction or thoracic correction are often performed to improve shoulder stability and quality of life (13, 14). In contrast, for patients with mild symptoms, preserved upper limb function, and adequate compensatory muscle strength, conservative management with regular follow-up may represent a more appropriate strategy, avoiding unnecessary surgical risks. This case provides longitudinal clinical observations extending to 12 years of age, including long-term follow-up after the diagnosis of CCD. Although the patient exhibited several characteristic skeletal manifestations of CCD, including bilateral clavicular pseudarthrosis, he maintained preserved shoulder function, normal muscle strength, and unrestricted participation in daily activities and sports throughout follow-up. No pain, recurrent fractures, neurological compromise, or deterioration in functional status was observed. These findings support a function-oriented management strategy, in which treatment decisions are guided primarily by functional status rather than by structural abnormalities alone. Consequently, conservative management was considered appropriate following multidisciplinary evaluation. Moreover,dental abnormalities are among the most characteristic manifestations of CCD and represent an important component of long-term disease management (4). Delayed exfoliation of primary teeth, delayed eruption of permanent teeth, and the presence of supernumerary teeth are frequently observed and may result in malocclusion or require multidisciplinary dental intervention (8). In this patient, dental involvement was characterized by delayed tooth eruption and delayed dental development without obvious supernumerary teeth or current indications for orthodontic or oral surgical treatment. Nevertheless, continued dental surveillance remains important because eruption abnormalities and occlusal problems may become more apparent during adolescence.This case highlights a function-oriented approach to clinical decision-making: in patients without pain, progressive deformity, neurovascular compromise, or functional limitation, particularly in the context of systemic skeletal dysplasia such as CCD, surgical intervention should be carefully considered with a thorough risk–benefit assessment. The absence of functional deterioration or major complications during long-term follow-up further supports the feasibility of conservative management in selected “function-preserved” patients.

Regarding growth and endocrine management, this patient had hypothyroidism diagnosed in infancy and received regular levothyroxine replacement therapy, with thyroid function monitored during follow-up. Before rhGH therapy, pediatric/endocrine follow-up showed a persistently low height percentile and delayed bone age despite adequate thyroid hormone replacement. Recombinant human growth hormone (rhGH) therapy was initiated at approximately 8 years of age after careful evaluation and counseling regarding potential risks and benefits. Evidence regarding rhGH therapy in CCD remains limited and inconsistent. Some reports have described improvement in growth velocity (15, 16), whereas others have suggested that the effect on final height remains uncertain (17). The biological basis for variable growth responses is also unclear. RUNX2 plays a central role in osteoblast differentiation and bone formation (18), while the GH/IGF-1 axis regulates skeletal growth through pathways such as JAK2/STAT5 (19). However, the interaction between RUNX2-mediated skeletal dysplasia and GH/IGF-1-related growth regulation remains incompletely understood. In this patient, height gain and height SDS improved during rhGH-related follow-up; however, given the single-patient nature of this report and the potential influences of hypothyroidism, normal developmental progression, pubertal maturation, and the underlying skeletal dysplasia, this finding should be regarded as observational of rhGH in CCD.

Given the marked phenotypic heterogeneity of CCD, similar radiographic abnormalities may be associated with different functional outcomes. In this patient, bilateral clavicular pseudarthrosis, mild pectus excavatum, and delayed dental development did not result in pain, progressive deformity, recurrent fractures, neurovascular compromise, respiratory symptoms, or restriction of daily activities during follow-up. These findings indicate that anatomical abnormalities alone should not determine the need for intervention. Instead, treatment decisions and family counseling should be based on functional status, quality of life, and longitudinal clinical changes. Conservative management remained appropriate in this patient, together with continued surveillance of growth, pubertal development, endocrine status, dental eruption and occlusion, shoulder function, clavicular stability, and potential skeletal complications. Surgical intervention may be reconsidered if pain, instability, progressive deformity, neurovascular compromise, respiratory impairment, or functional limitation develops. Genetic counseling and transition to adult multidisciplinary care should also be considered during long-term follow-up.

This study has several limitations. First, as a single case, the findings cannot be generalized to all carriers of the p.R211W variant. Second, the effect of rhGH therapy cannot be directly compared with untreated controls.

In summary, this case provides longitudinal evidence of the clinical course of genetically confirmed CCD associated with the RUNX2 p.R211W variant. Despite typical skeletal and dental manifestations, the patient maintained favorable functional status under individualized, function-oriented conservative management. Height SDS improved during rhGH-related follow-up; however, this finding should be interpreted cautiously because the effects of thyroid hormone replacement, normal growth progression, pubertal development, and the underlying skeletal dysplasia cannot be fully separated. This case highlights the importance of long-term multidisciplinary follow-up, careful endocrine evaluation, dental and orthopedic surveillance, and individualized selection of surgical intervention. Larger longitudinal studies are needed to better define the natural history, functional outcomes, and optimal management strategies for patients with CCD.

## Data Availability

The datasets presented in this article are not readily available because of ethical and privacy restrictions. Requests to access the datasets should be directed to the corresponding author.
